# Transformative Bioactive Wear Resistant Ti_3_Au:N and Ti_3_Au:O Coatings for Medical Implants and Devices

**DOI:** 10.1002/adhm.202503441

**Published:** 2025-10-30

**Authors:** Cecil Cherian Lukose, Ioannis Anestopoulos, Iraklis‐Stavros Panagiotidis, Guillaume Zoppi, Anna M Black, Lynn G. Dover, Leon Bowen, Ángel Serrano‐Aroca, Lorenzo Mendola, Davide Morrone, Mihalis I. Panayiotidis, Martin Birkett

**Affiliations:** ^1^ Faculty of Engineering and Environment Northumbria University Newcastle upon Tyne NE1 8ST UK; ^2^ Department of Cancer Genetics Therapeutics and Ultrastructural Pathology The Cyprus Institute of Neurology and Genetics Nicosia 1683 Cyprus; ^3^ Faculty of Health and Life Sciences Northumbria University Newcastle upon Tyne NE1 8ST UK; ^4^ Department of Physics G.J. Russell Microscopy Facility Durham University Durham DH1 3LE UK; ^5^ Biomaterials and Bioengineering Lab Department of Biotechnology Universidad Católica de Valencia San Vicente Mártir c/Guillem de Castro 94 Valencia 46001 Spain; ^6^ Translational Research Centre San Alberto Magno Universidad Católica de Valencia San Vicente Mártir, c/Quevedo 2 Valencia 46001 Spain; ^7^ Nanovea Inc 6 Morgan Ste 156 Irvine CA 92618 USA; ^8^ Department of Comparative Biomedical Sciences, College of Veterinary Medicine Mississippi State University 240 Wise Center Drive Starkville MS 39762 USA

**Keywords:** antimicrobial, biocompatible, high hardness, Ti_3_Au thin film coating, wear resistant

## Abstract

Current biomedical implants have high mechanical strength and corrosion resistance, but are highly susceptible to wear and lack antimicrobial properties to fight infection. Here, nitrogen and oxygen‐doped Ti_3_Au structures are successfully grown with increased dislocation slip plane energy and α‐phase stability for the first time to achieve exceptional biotribological and antibacterial performance. The new Ti_3_Au:N and Ti_3_Au:O coatings are grown on Ti‐6Al‐4 V substrates via magnetron co‐sputtering of Ti/Au in Ar:N_2_ and Ar:O_2_ environments at 450 °C and characterized for structural, chemical, tribomechanical, biocompatibility, and antibacterial properties. The reactive gas environments produce highly crystalline Ti_3_Au:N and Ti_3_Au:O coatings containing α‐Ti_3_Au phases, with partial columnar structures. All coatings present extremely safe cytotoxicity profiles with leached ion concentrations <0.2 ppm, and antibacterial performance like pure Cu and Ag, with a drastic reduction in bacterial colonies within 20 min of exposure. Nitriding and oxidizing the Ti_3_Au coating produces superhard scratch‐resistant surfaces with wear rates >20 times lower than the Ti‐6Al‐4 V substrate. The findings demonstrate new biocompatible Ti_3_Au:N and Ti_3_Au:O coatings with outstanding multifunctional biotribological and antibacterial properties with the potential to significantly extend the lifetime of medical implants and devices, such as joint replacements, bone screws, and surgical devices.

## Graphical abstract

Coatings of titanium‐gold are grown in nitrogen and oxygen environments via magnetron sputtering to simultaneously enhance their biotribological and antibacterial properties. The coatings are highly crystalline with superhard scratch‐resistant surfaces and wear rates 20 times lower than the bare Ti‐6Al‐4V substrate. All coatings present excellent biocompatibility and antibacterial performance like pure Cu and Ag.

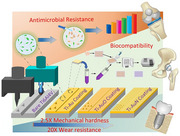

## 1 Introduction

There is growing interest in advanced biocompatible coatings with multifunctional properties to enhance the performance of medical and dental devices and components and improve patient outcomes and quality of life. Coatings made from metals, ceramics, polymers, and their hybrid combinations are attracting continuous attention in a wide range of biomedical applications such as orthopedic and orthodontic implants, fracture fixations and plates, stents, sensors, and drug delivery systems, to enhance key device performance properties such as biocompatibility, antimicrobial potency, osseointegration, corrosion resistance, and wear resistance.^[^ ^1^, ^2^, ^3^ ^]^

Two of the most popular biomaterials that have benefited from the application of surface coatings are Ti‐6Al‐4 V, and CoCrMo. These alloys are used extensively in the manufacture of medical and dental devices due to their excellent mechanical strength, corrosion resistance, and superior osteointegration.^[^ ^4^, ^5^ ^]^ However, issues surrounding their relatively low mechanical wear resistance and health concerns over leaching of potentially toxic metal ions still prevail as significant limitations of these alloys.^[^ ^6^, ^7^, ^8^ ^]^ Numerous advanced transition metal‐based Ti, Ta, Zn, Zr, Cr, Nb^[^ ^6^, ^8^, ^9^, ^10^, ^11^, ^12^, ^13^ ^]^ and diamond‐like carbon (DLC)^[^ ^14^ ^]^ coating systems have been developed to coat Ti‐6Al‐4 V and CoCrMo medical devices to overcome these issues while preserving the mechanical integrity of the underlying bulk alloy. Of these, Ti‐based coatings are by far the most widely used due to their excellent durability, fatigue resistance, corrosion resistance, non‐toxic properties, and proven compatibility with Ti‐6Al‐4 V and CoCrMo substrates. Although pure Ti coatings are relatively soft and offer limited performance improvement over that of the base alloy, incorporating other elements into the coating matrix during the deposition process can have a profound effect on the overall coating structure and resulting properties. Two of the most widely studied elements are nitrogen (N) and oxygen (O), which are doped into the growing Ti coating matrix in various quantities to form Ti─N and Ti─O coatings with superior mechanical properties, including high strength, toughness, hardness, and wear resistance, as well as excellent biocompatibility and chemical stability. However, despite these advanced properties, the application of Ti─N and Ti─O coatings to medical devices remains cautious due to concerns over pinholes, potential delamination, cohesive failure, and generation of ceramic wear particles.^[^ ^7^, ^8^, ^15^ ^]^

An alternative Ti‐based material that is receiving increasing attention is the TiAu intermetallic due to its excellent biocompatibility and superior mechanical performance.^[^ ^16^, ^17^, ^18^, ^19^, ^20^, ^21^, ^22^ ^]^ Recent studies have confirmed that the outstanding biocompatibility of the individual Ti and Au elements is preserved when the two combine to form TiAu intermetallics. Extensive testing shows that TiAu coatings are highly biocompatible across the entire Ti_x_Au_(1‐x)_ chemical composition range, with L929 mouse fibroblast cell viability values of >90% following 168 h of both direct and indirect exposure.^[^ ^21^ ^]^ Recent research has also demonstrated a non‐monotonous increase in hardness of TiAu coatings deposited on Ti‐6Al‐4 V substrates, with increasing Au concentration, reaching a peak value >12 GPa at ≈25 at.% Au, with the formation of the cubic β‐Ti_3_Au compound.^[^ ^19^ ^]^ This sudden increase in hardness is 2–3 times higher than the values of ≈3.37 GPa and 5.02 GPa recorded for Ti‐6Al‐4 V and CoCr alloys^[^ ^23^, ^24^ ^]^ and is caused by the higher valence electron density of Au compared to Ti, which leads to increased bond length, as well as denser packing of the 14‐fold β phase unit cell compared to the 12‐fold α phase unit cell, which presents a higher energy barrier to dislocation plane slipping in the lattice system. However, while these initial studies on the β‐Ti_3_Au structure have demonstrated its higher hardness and biocompatibility equivalent to its individual constituents, its mechanical and tribological performance could be transformed through the addition of interstitial elements like N and O to raise the energy barrier to dislocation plane slipping.

Here, we report on novel Ti_3_Au:N and Ti_3_Au:O biomaterial coatings with exceptional biotribological and antibacterial potential. The thin film coatings are grown on Ti‐6Al‐4 V substrates via magnetron co‐sputtering of Ti and Au elements in reactive N_2_ and O_2_ gas environments at elevated temperature and subsequently characterized for their structural, chemical, morphological, mechanical, tribological, biocompatibility, and antibacterial properties. We demonstrate new Ti_3_Au:N and Ti_3_Au:O biomaterials with outstanding biocompatibility, wear resistance 20 times higher than Ti‐6Al‐4 V alloy and antibacterial performance equal to pure Cu and Ag, with the potential to coat and protect a range of medical and dental devices to extend their lifetime in the human body.

## 2 Results and Discussion

### 2.1 Structural, Chemical, and Morphological Properties

**Figure 1:**
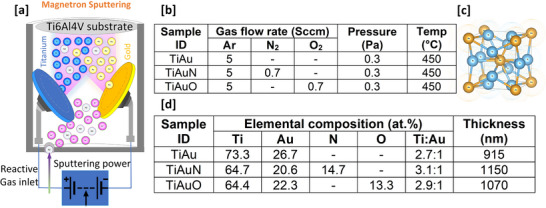
a) Schematic of the reactive magnetron sputtering process to grow TiAu, TiAuN, and TiAuO thin films. b) Table highlighting sputtering process parameters to achieve TiAu, TiAuN, and TiAuO thin films. c) Schematic representation of the location of Ti (blue) and Au (orange) atoms within a β‐Ti_3_Au unit cell. d) Table highlighting the film thickness (±22 nm thickness error, *n* = 5) and elemental composition (±0.3 at% error, *n* = 5) of the TiAu, TiAuN, and TiAuO thin films.

Results for EDX chemical composition and thickness of the TiAu, TiAuN, and TiAuO thin films are presented in **Figure** **1**d. The stoichiometric ratio of Ti:Au is within 0.3 of the required 3:1 ratio for all three thin film samples, while the levels of N and O in the TiAuN and TiAuO films have similar values of 14.7 and 13.3 at.%, respectively. The thickness of the three film samples is also in a similar range of ≈900–1100 nm. The N and O‐doped films are slightly thicker than the undoped TiAu film due to an increase in sputter rate caused by the addition of the reactive gas.^[^ ^25^ ^]^

**Figure 2:**
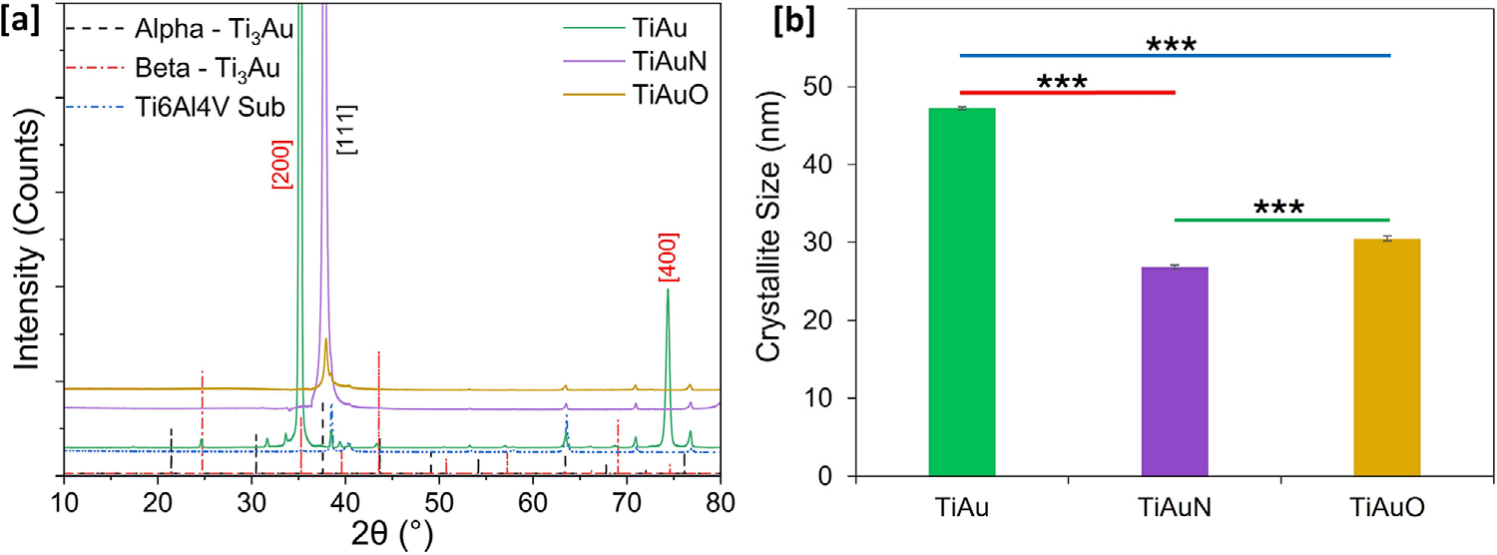
a) XRD patterns of TiAu, TiAuN, and TiAuO samples deposited on Ti‐6Al‐4 V substrates. b) Crystallite size calculated for TiAu, TiAuN, and TiAuO samples using Scherrer's equation. Data presented as mean ± SD, *n* = 3. Statistically significant differences in results are indicated as *p < 0.05, **p < 0.01, and ***p < 0.001.

Diffraction patterns for the TiAu, TiAuN, and TiAuO thin films deposited on Ti‐6Al‐4 V substrates are presented in **Figure** **2**a. The Ti_3_Au intermetallic exists in two different phases, denoted as α‐phase and β‐phase. Both these phases are cubic structures, but the α‐phase is formed at lower temperatures with a 221 space group and Pm3m symmetry, whereas the β‐phase develops at temperatures above 350–400 °C with a 223 space group and Pm3n symmetry.^[^ ^19^ ^]^ The pattern for the TiAu sample (green line) shows an excellent match to the β‐Ti_3_Au intermetallic (ICSD 58605—red dotted line) with preferential orientation along the [200] directions, with dominant peak at 35.17°. However, when N and O are doped into the TiAu film, there is a sudden transformation from β‐phase to α‐phase Ti_3_Au, with both TiAuN (violet line) and TiAuO (yellow line) samples displaying a single sharp peak at 37.78° and 37.94°, which is characteristic of the [111] oriented α‐Ti_3_Au intermetallic [ICSD 58604—black dotted line]. Elements like N and O form interstitial solid solutions where the solute atoms occupy space in between the interstices in the Ti atom, and at this concentration they are known to be α‐phase stabilizers.^[^ ^26^ ^]^ These interstitials dissolve easily in α‐Ti at concentrations below 15 at.%^[^ ^27^ ^]^ and could therefore be increasing the β‐phase formation temperature. Previous studies have demonstrated that α‐Ti can accommodate significant concentrations of interstitial elements such as O and N under carefully controlled deposition conditions. Kværndrup et al.^[^ ^28^ ^]^ reported that when the partial pressure of reactive gases (O_2_ or N_2_) is maintained at extremely low levels within an inert argon carrier atmosphere, and the substrate temperature is elevated (e.g., 500 °C), interstitial doping is thermodynamically favoured. Under these conditions, the inward diffusion flux of interstitial species into the Ti matrix exceeds the rate of surface oxidation or nitridation, enabling the formation of metastable solid solutions without secondary phase formation. In previous studies, solid solution incorporation of up to 30 at.% O was achieved in 75 µm‐thick α‐Ti foil without the formation of oxide phases.^[^ ^28^ ^]^ In the current work, sputter deposition of Ti_3_Au thin films (≈1 µm thick) was carried out under low partial pressures of O_2_ and N_2_ in an Ar environment at low deposition pressure (>0.3 Pa) and a substrate temperature close to 500 °C. These conditions enabled the incorporation of higher atomic concentration (≈15 at.%) of O or N into the Ti_3_Au matrix. Despite the high interstitial content (≈15 at.%), XRD analysis did not reveal any peaks corresponding to titanium oxides or nitrides, suggesting successful formation of an interstitial solid solution. These results show that thinner films of a ternary system deposited with careful control of gas‐phase chemistry and thermal conditions can enable substantial interstitial doping of α stabilizers in the Ti matrix.

The mean crystallite sizes for each of the three thin film samples, calculated from their dominant diffraction peak using Scherrer's equation^[^ ^29^ ^]^ are presented in Figure 2b. The mean crystallite size of the TiAu thin film is estimated to be 47.2 nm, which decreases significantly to 26.8 and 30.5 nm for the TiAuN and TiAuO films, respectively. This reduction is attributed to a distinct phase transformation from the β‐phase to the α‐phase of Ti_3_Au, as observed in the X‐ray diffraction patterns (Figure 2a). The lattice spacing (d) and lattice parameter (a) were calculated using Bragg's Law. For the standard β‐Ti_3_Au phase, the (200) reflection at 2θ = 35.17° corresponds to a d‐spacing of 2.55 Å and a lattice parameter of 5.10 Å. In contrast, the TiAuN sample exhibits a (111) peak at 2θ = 37.78°, corresponding to a d‐spacing of 2.37 Å and a lattice parameter of 4.12 Å. Similarly, the TiAuO film shows a (111) peak at 2θ = 37.94°, with a d‐spacing of 2.36 Å and a lattice parameter of 4.10 Å. These results demonstrate a clear reduction in unit cell size, indicating that N and O doping effectively stabilize the α‐phase structure in TiAu‐based thin films. The interstitial doping induces the β‐to‐α phase transformation, which promotes the formation of smaller densely packed crystallites and consequently reduces the average crystallite size. Further, the addition of N and O interstitial species act as pinning sites at the grain boundaries, leading to the grain refinement.^[^ ^30^, ^31^, ^32^ ^]^ According to the Hall‐Petch relationship, smaller refined grains offer larger grain boundaries, which will in turn act as a barrier to the dislocation movement under an external force, resulting in increased hardness of material.

**Figure 3:**
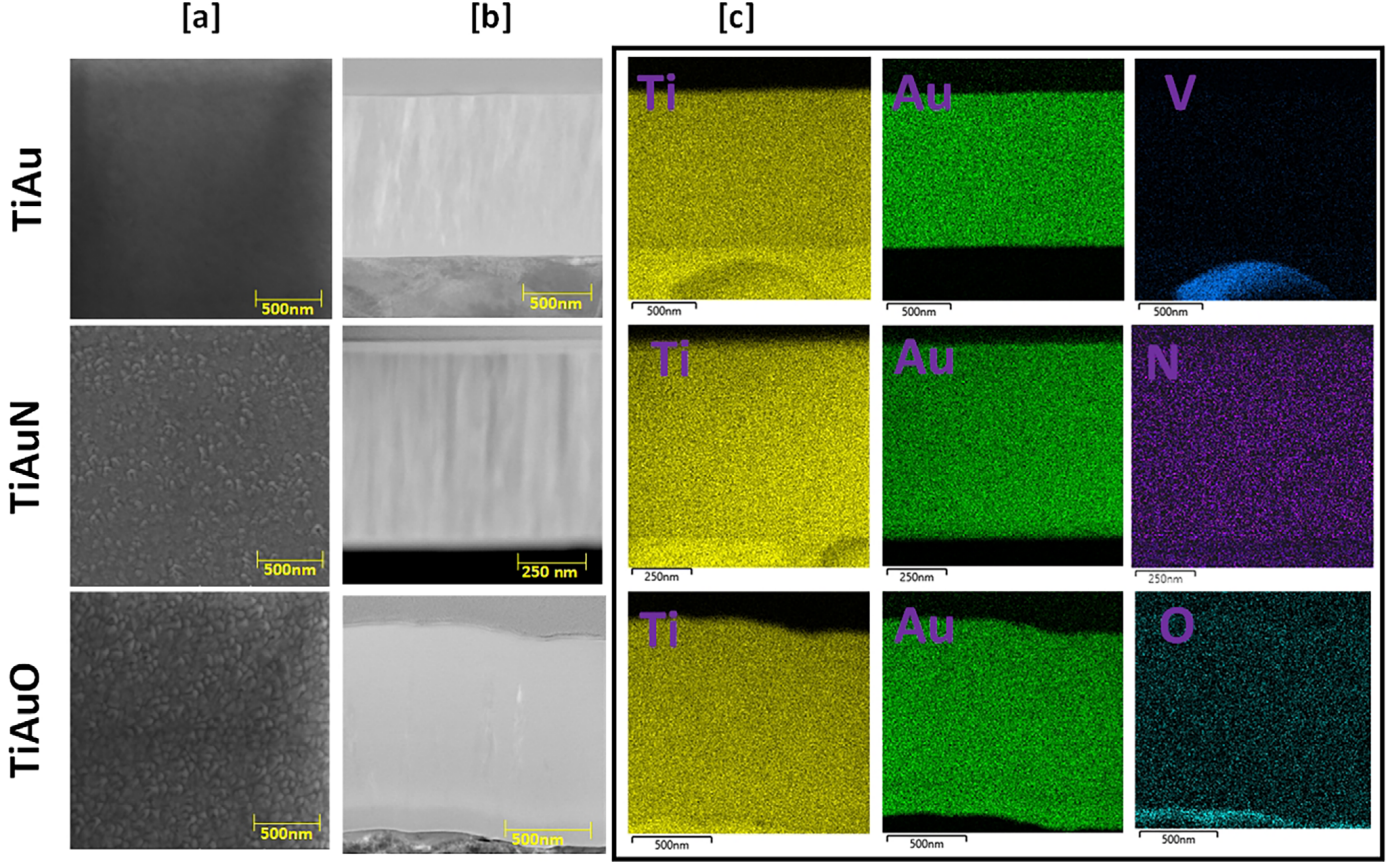
a) SEM surface images of TiAu, TiAuN, and TiAuO thin films. b) TEM cross‐section images of TiAu, TiAuN, and TiAuO thin films. c) Cross‐sectional elemental mapping showing the distribution of Ti, Au, and dopant elements across the film thickness of TiAu, TiAuN, and TiAuO thin films.

Surface SEM images of the TiAu, TiAuN, and TiAuO thin films presented in **Figure** **3**a show that the surface of TiAu film is extremely smooth and dense, with no visible grains dispersed on the surface, signifying the development of well‐oriented β‐Ti_3_Au crystals.^[^ ^20^ ^]^ The presence of pure β phase is also supported by the XRD pattern for this sample (Figure 2a). This high‐quality structure is characteristic of Ti_3_Au thin films grown at elevated substrate temperature and low sputtering pressure, where higher energy incoming species and thermally driven surface diffusion lead to a densely packed columnar structure and smoother film surface.^[^ ^19^ ^]^ The presence of the α‐phase is verified in the diffraction patterns for the TiAuN and TiAuO samples (Figure 2a). TEM cross‐section images of the thin films are presented in Figure 3b, correlating with the film structures observed in the SEM images (Figure 3a). The TEM cross sections show that, unlike the columnar structure of TiAu, the addition of N and O to the TiAu film leads to the development of a tapered or partial columnar structure, which is representative of the α‐Ti_3_Au structure.

**Figure 4:**
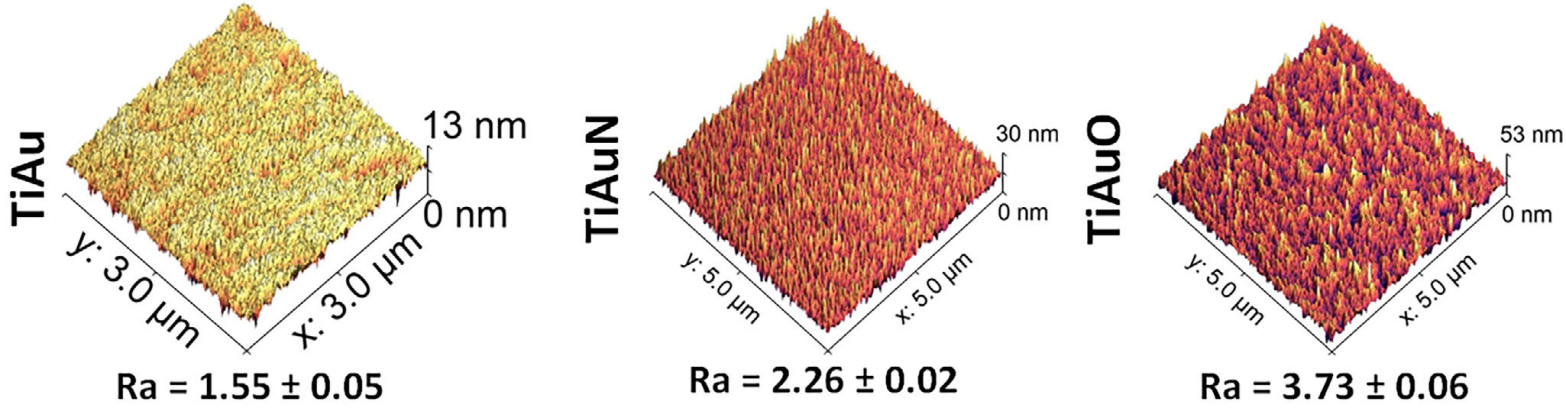
AFM surface maps of TiAu, TiAuN, and TiAuO thin films, showing feature heights and average surface roughness (R_a_) values, presented as mean ± SD, *n* = 5.

The elemental maps of the cross‐sectional TEM images are presented in Figure 3c, and track the distribution of Ti, Au, V, O, and N elements through the TiAu, TiAuN, and TiAuO thin films and Ti‐6Al‐4 V substrate thickness. The presence of V and the increased intensity of Ti in the TiAu sample correlate with the film‐substrate interface. All three samples, TiAu, TiAuN, and TiAuO present a uniform distribution of Ti and Au across the film depth. However, N and O, being light elements are inherently difficult to resolve using elemental mapping via EDX techniques. Their weak X‐ray signals, combined with the absorption of low‐energy X‐rays by the sample and overlapping spectral peaks from heavier elements, significantly hinder accurate detection. It is well established that N and O exhibit high solubility in α‐Ti, up to 33.3 at% for N and 22.0 at% for O, where they exist as interstitial solid solutions, contributing to surface hardening. Due to these detection challenges, the TiAuN films were analyzed using a smaller field of view to better reveal the subtle reduction in N content at the boundaries. In contrast, for the TiAuO films, an increased O concentration is clearly visible at the film–substrate interface. This elevated initial surface O content results from the strong affinity of Ti in the Ti6Al4V substrate toward O gas in the reactive deposition environment. However, when the deposition process commences and the film begins to grow, O becomes more uniformly distributed due to its continued interaction with the Ti species within the growing film. As seen in section 2.2, this preferential oxidation at the interface is not significant enough to affect film adhesion during wear testing or compromise the mechanical properties of the thin film surface. This change in film structure between undoped and doped TiAu films leads to an increase in surface texture, which is clearly visible in the AFM images in **Figure** **4**, where the maximum feature height and average surface roughness (R_a_) increases from 13 and 1.55 nm for the TiAu film to 30 and 2.26 nm for the TiAuN film and 53 and 3.73 nm for the TiAuO film.

### 2.2 Mechanical and Tribological Properties

The mechanical and tribological performance of TiAu, TiAuN, and TiAuO thin films deposited on

**Figure 5:**
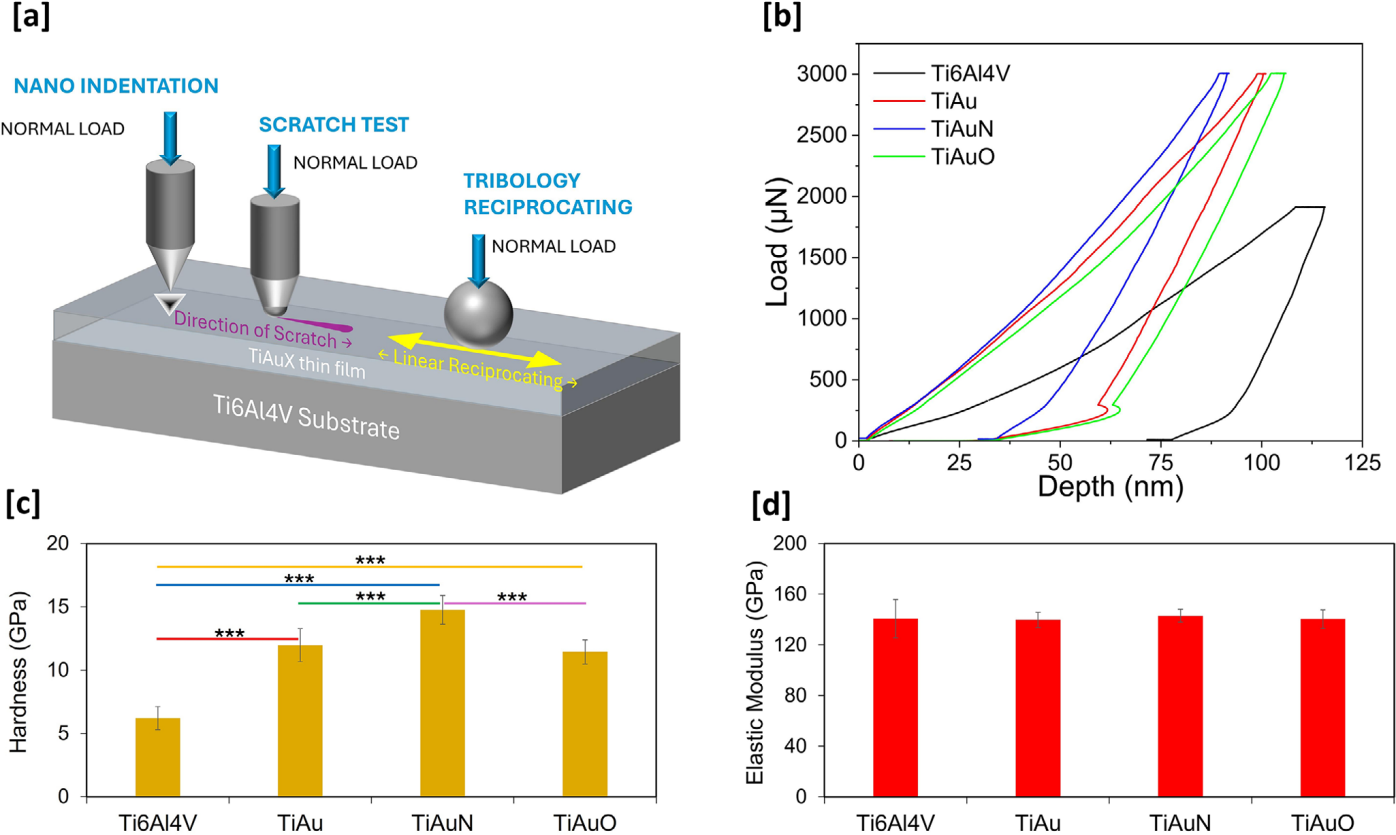
a) Schematic representation of the nanoindentation, scratch, and tribological wear test procedures. Results for mechanical characterization performed on the TiAu, TiAuN, and TiAuO thin films and Ti‐6Al‐4 V substrates. b) Load‐displacement curves showing performance improvement of the thin films compared to bare Ti‐6Al‐4 V substrate. Plots of c) mechanical hardness, and d) reduced elastic modulus, extracted from load‐displacement curves. Data presented as mean ± SD, *n* = 16. Any statistically significant differences in results are indicated as *p < 0.05, **p < 0.01, and ***p < 0.001.

Ti‐6Al‐4 V substrates, were studied using nanoindentation, scratch test and linear reciprocating wear test, see **Figure** **5**a. The results from the thin films are compared to bare, uncoated Ti‐6Al‐4 V substrates to highlight the drastic performance improvements these novel coatings can bring.

The nanoindentation load‐displacement curves (Figure 5b) are smooth and continuous, without any sudden displacement variation along the X‐axis, further supporting the XRD observations relating to the absence of major phase differences. The homogenous nature of the films will also aid their adhesion to the substrate surface. For the bare Ti‐6Al‐4 V substrate, the tip of the indenter penetrates to a maximum depth (h_max_) of 116 nm with a lower peak load of 1900 µN, before returning to a contact depth of 99 nm during the unloading section of the curve. Whereas at the peak load of 3000 µN, the tip only achieves a maximum penetration depth of 102 nm on the TiAu thin film, before elastically recovering to a contact depth of 71 nm. Under the same testing conditions, the maximum displacement is limited to 92 nm for the TiAuN thin film and recovers to 61 nm, evidencing an increase in hardness for these films. While for the TiAuO thin film, the maximum penetration depth increases slightly to 107 nm and recovers to 77 nm, demonstrating a slight reduction in hardness when compared to the TiAu and TiAuN films. The region representing the elastic recovery and plastic deformation is very similar for all three samples and suggests that the elastic modulus of these films remains within close proximity.

The average values of mechanical hardness and reduced elastic modulus for the TiAu, TiAuN, and TiAuO thin films and Ti‐6Al‐4 V substrate are presented in Figure 5c,d, respectively. There is a significant increase in hardness for the thin films of 199–246% when compared to the bare Ti‐6Al‐4 V substrate. The average hardness of the Ti‐6Al‐4 V substrate was measured at 6.2 GPa, which is similar to previously reported values^[^ ^33^ ^]^ and rapidly increases to 12 GPa for the TiAu thin film. The addition of N in the TiAuN thin film produces a further increment in hardness to an average value of 14.7 GPa, while addition of O to form the TiAu‐O film does not produce any noticeable change in hardness compared to the TiAu film. The average reduced elastic modulus values are comparable throughout the entire sample range, with error bars lower than 7.3 GPa for each point, except for the bare Ti‐6Al‐4 V substrate, which records higher error bars of 15.2 GPa due to its higher surface roughness. The thin films show elastic modulus results similar to the bare Ti‐6Al‐4 V substrates with average values of 139–143 GPa. It is of interest that the TiAuN thin film coating can increase mechanical hardness by up to 246% compared to the bare Ti‐6Al‐4 V, while maintaining a similar value of reduced elastic modulus. While the solid solution of interstitial N drives up the hardness of the TiAuN thin film, the presence of softer Au helps to reduce the brittleness of the resulting mixture. This result is similar to that of Kvaerndrup et al. who observed a spectacular increase in hardness with increasing interstitial contents of O and N of up to 40 at.% in single‐phase α‐Ti thin film foils.^[^ ^28^ ^]^ They reported similar values of 12–14 GPa for hardness and 120–140 GPa for modulus at O/N concentrations of 15–20 at.% and suggested that interstitial hardening provides a means to increase the surface hardness of α‐Ti without TiN formation. However, the interstitial content should be kept below 17 at.% to avoid severe embrittlement.

**Figure 6:**
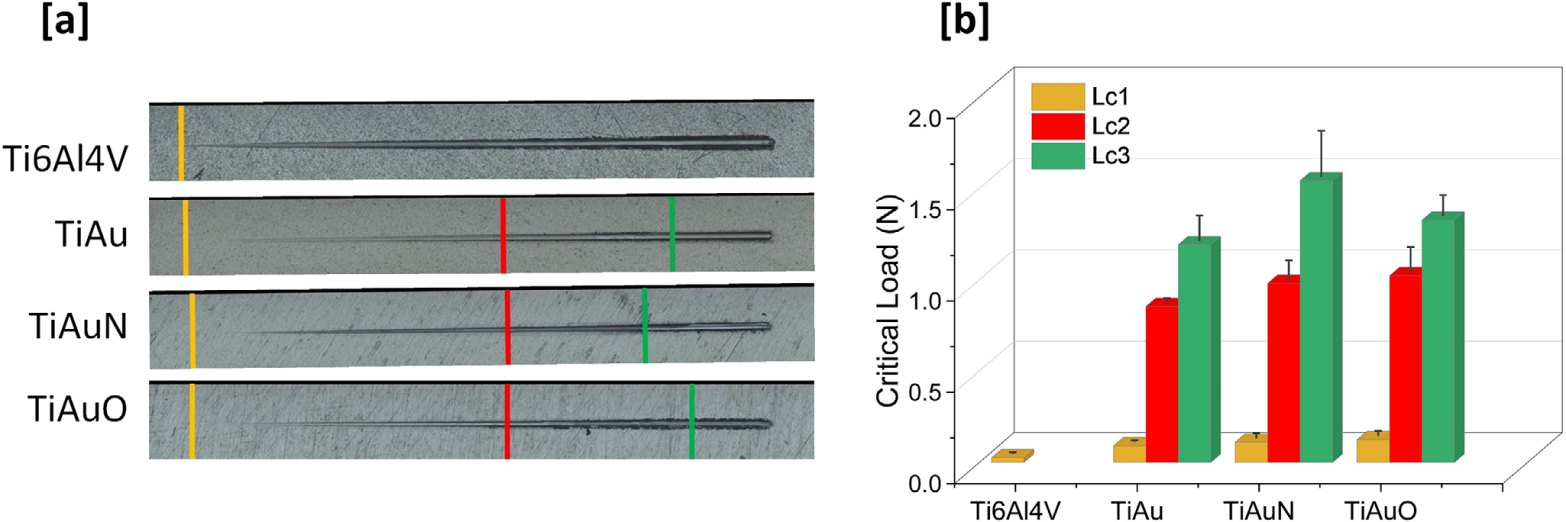
Results for scratch testing performed on the TiAu, TiAuN, and TiAuO thin films and Ti‐6Al‐4 V substrates. a) Optical images of scratch tests, and b) Plot of scratch test critical loads. Data presented as mean ± SD, *n* = 3.

Images of superficial scratches on the TiAu, TiAuN, and TiAuO thin films and Ti‐6Al‐4 V substrate surface and corresponding values of critical loads (Lc1, Lc2, and Lc3) are presented in **Figure** **6**a,b, respectively. Lc1 indicates the initiation of a visible track, which is mainly located at the beginning of the scratch track, Lc2 is assigned to the first cohesive failure or first chipping point, which mostly displays spalling failure mode or internal cracks, and Lc3 is defined as the critical load where the thin films show a complete adhesive failure exposing completely the Ti‐6Al‐4 V substrate, (before Lc3, the coating remains mostly intact).^[^ ^34^ ^]^ As expected, the Ti‐6Al‐4 V sample requires the lowest scratch normal force of 0.026 N to initiate a visible failure (Lc1) on its surface. This force increases by ≈3.5–4.5 times to values of 0.09, 0.11, and 0.12 N for the TiAu, TiAuN, and TiAuO thin films, respectively, representing a similar increase to that observed for hardness (Figure 5c). The first indication of thin film adhesion failure from the Ti‐6Al‐4 V substrate (Lc2) is observed at a force of 0.85 N for the TiAu coating and increases to values of 0.98 and 1.02 N for the TiAuN and TiAuO thin films, respectively. However, the critical load required to completely peel off the coating from the Ti‐6Al‐4 V substrate (Lc3) is higher for the TiAuN thin film (1.54 N), when compared to the TiAu (1.19 N) and TiAuO (1.33 N) samples. This trend was also observed in the scratch images, where the TiAuN sample displayed minimal signs of film spalling and peeling compared to the TiAu and TiAuO thin films. These results suggest a good correlation between thin film hardness (Figure 5c) and scratch resistance (Figure 6b).

**Figure 7:**
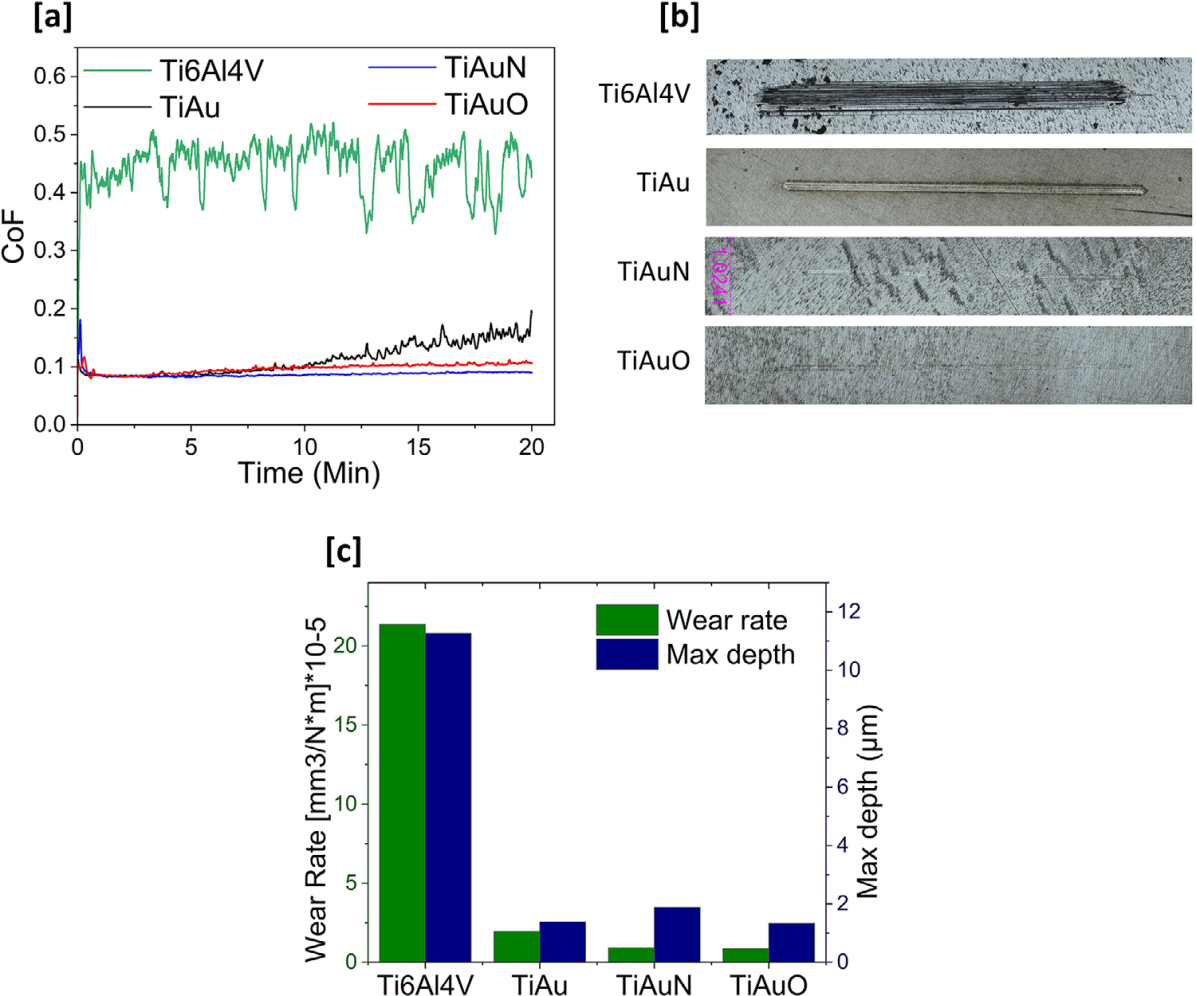
Results for wear testing performed on the TiAu, TiAuN, and TiAuO thin films and Ti‐6Al‐4 V substrates in SBF. a) Friction coefficient curves, b) Optical images of wear scars, and c) Specific wear rates and maximum wear scar depths.

Results for linear reciprocating wear testing of the TiAu, TiAuN, and TiAuO thin films and Ti‐6Al‐4 V substrate sliding against stainless steel balls at 1.52 N normal load in SBF are presented in **Figure** **7**. As shown in Figure 7a, the friction coefficient of the Ti‐6Al‐4 V substrate is much higher and more erratic than that of the thin films, fluctuating between 0.4 and 0.5 throughout the 20‐min test duration. Coating the substrate with the TiAu thin film significantly reduces the friction coefficient to ≈0.1 for the first 10 min of sliding before gradually increasing to ≈0.2 after 20 min. The addition of O and N to the TiAu thin film gives further reductions in friction coefficient, with values of 0.09 and 0.1 recorded for the TiAuN and TiAuO samples, respectively, throughout the full 20 min test duration. This significant reduction in friction coefficient of the thin films compared to the Ti‐6Al‐4 V substrate can be related to their higher surface hardness (Figure 5c) and scratch resistance (Figure 6b), which leads to a reduction in real contact area with the stainless‐steel counter body and a corresponding reduction in surface plastic removal.^[^ ^35^, ^36^ ^]^ These results for the friction coefficient are supported by the wear track surface images in Figure 7b and corresponding values of maximum wear track depth and wear rate in Figure 7c. The wear track on the Ti‐6Al‐4 V substrate is ≈300 µm wide with a maximum depth of 11.3 µm, which results in a wear rate of ≈215 × 10^−6^ mm^3^ Nm^−1^. By comparison, the wear track width and maximum depth on the TiAu thin film are <100 and 1.4 µm, respectively, which leads to a >10‐fold reduction in wear rate to <20 × 10^−6^ mm^3^ Nm^−1^. This performance is improved even further with the addition of O and N into the TiAu coating matrix. The wear tracks on the TiAuN and TiAuO thin films are almost invisible, with wear rate values <10 × 10^−6^ mm^3^ Nm^−1^, which is half that of the TiAu thin film and over 20 times lower than the wear rate of the Ti‐6Al‐4 V substrate. This significant decrease in wear rate is due to the strengthening effect and reduction of the friction coefficient of the TiAu thin films, which is further enhanced through nitriding and oxidizing to form TiAuN and TiAuO thin film structures^[^ ^37^, ^38^ ^]^ with α‐TiAu stabilizing phase. Overall, the results presented in this section confirm that coating Ti‐6Al‐4 V substrates with TiAu, TiAuN, and TiAuO thin films can significantly enhance their mechanical and tribological performance.

### 2.3 Biological Properties

#### 2.3.1 Biocompatibility‐Cytotoxicity Test Results

**Figure 8:**
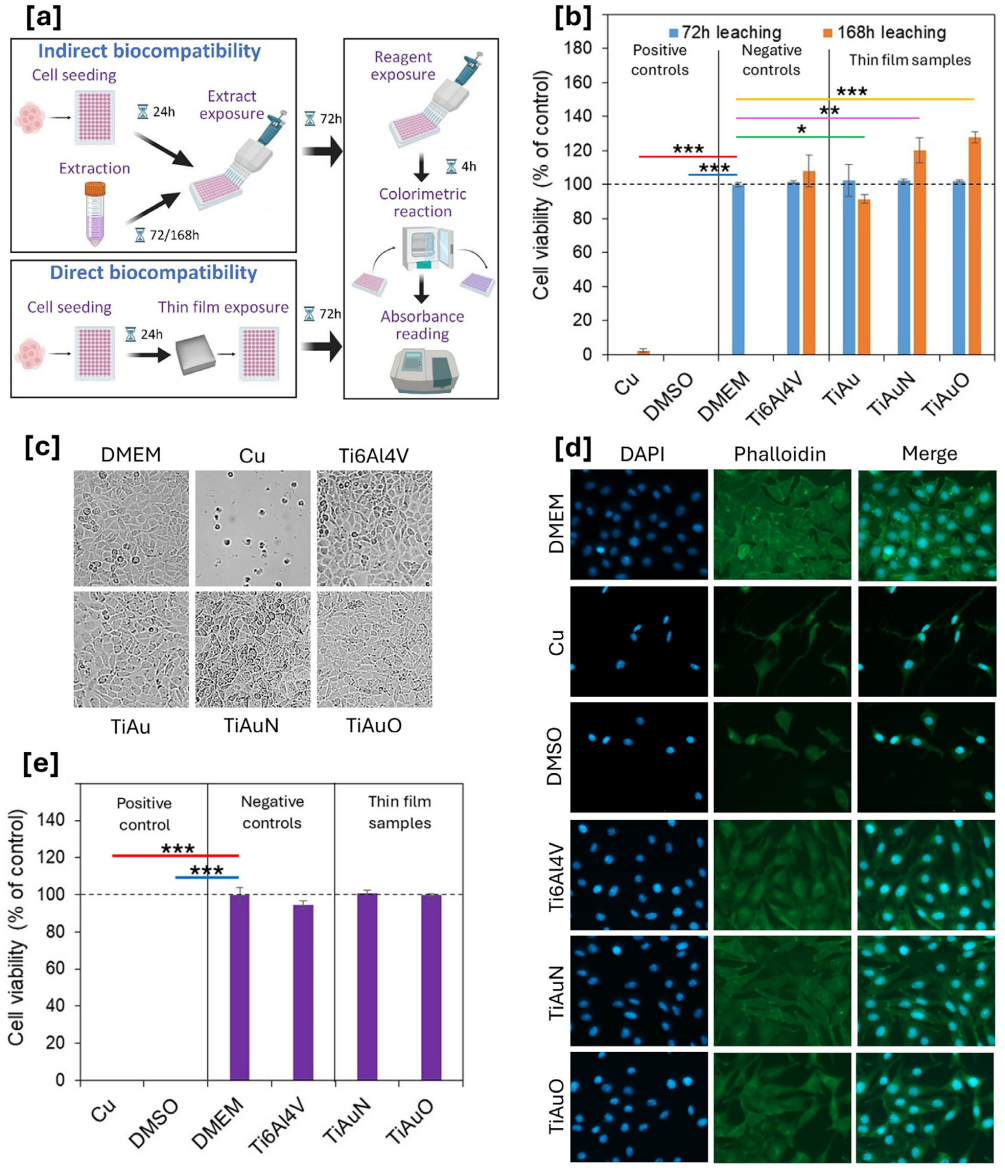
a) Schematic representation of both indirect (upper panel) and direct (lower panel) exposures of L929 fibroblast cells with TiAu, TiAuN, and TiAuO thin films, followed by determination of cytotoxicity/biocompatibility using the Alamar Blue assay. b) Viability levels of L929 cells after 72 h incubations with 72 and 168 h thin film leached extracts, compared to negative (Ti‐6Al‐4 V and DMEM) and positive (Cu and 10% DMSO). c) Morphological changes of L929 cells following 72 h exposures with 168 h leached extracts from thin films, Cu, and Ti‐6Al‐4 V, compared to untreated (DMEM) cells. d) Immunofluorescence images of L929 cells following direct exposures with Cu, 10% DMSO (positive controls), Ti‐6Al‐4 V (negative control) and thin films, cells were fixed and stained with DAPI (first panel, blue color) and anti‐Phalloidin (second panel, green color), in order to detect changes on their morphology and actin cytoskeleton organization. Merged images for DAPI/Phalloidin staining are shown in the third panel. e) Viability levels of L929 cells after 72 h direct incubations with thin films, compared to negative (Ti‐6Al‐4 V and DMEM) and positive (Cu and 10% DMSO) controls. Data presented as mean ± SD, *n* = 3. Any statistically significant differences in results are indicated as *p < 0.05, **p < 0.01, and ***p < 0.001.

The biocompatibility/cytotoxicity of the TiAu, TiAuN, and TiAuO thin films were assessed by means of both direct and indirect exposures against L929 fibroblast cells using the Alamar blue assay (**Figure** **8**a). Results of L929 cell viability following indirect exposures of both 72 and 168 h leached thin film extracts against L929 cells for 72 h are presented in Figure 8b. Specifically, both Cu substrate extract and 10% DMSO solution used as positive controls exhibited a significant cytotoxic effect with a dramatic reduction in cell viability levels. Conversely, the Ti‐6Al‐4 V substrate extract was shown to possess a safe cytotoxic profile against fibroblasts, since viability levels were comparable (≥100%) to those of DMEM (blank sample) following leaching periods of both 72 and 168 h. Similarly, all tested thin film extracts showed excellent cell viability levels of ≥100% following 72 h of leaching. There was a slight reduction in cell viability to 92% for the TiAu sample after the extended leaching period of 168 h, whereas the TiAuN and TiAuO samples were found to increase/stimulate cell viability levels above 120%. This slight decline in cell viability for the TiAu sample following 168 h of leaching could be caused by an increase in concentration/release of metal ions during the prolonged period. However, the result is still above 90%, suggesting that the sample is non‐cytotoxic and highly biocompatible. The above response pattern, following indirect exposures, was also further supported by observation of L929 cells’ morphology (Figure 8c). Specifically, microscopic observation of fibroblasts under different exposures indicated a stark difference in cell morphology between the Cu substrate (positive control) versus the Ti‐6Al‐4 V substrate (negative control) and the thin films. For instance, Cu substrate exposures resulted in a significant cell shrinkage and death, indicative of its strong cytotoxic effect, ultimately resulting in confluency reduction. Whereas, both morphology and confluency of cells exposed to the Ti‐6Al‐4 V substrate and TiAu, TiAuN, and TiAuO thin films, were unaffected and comparable to those of DMEM (blank sample), supporting a safe cytotoxic profile of these samples. Furthermore, the concentration of ions leached from the TiAu, TiAuN, and TiAuO thin films deposited on Ti‐6Al‐4 V substrates was measured using ICP‐OEMS. Overall, the analysis did not detect any significant levels of ions following both 72 and 168 h leaching periods. After 72 h of leaching, only 0.07 ppm of Au ions were present, whereas Ti and Al ions were only detected after 168 h of leaching, with maximum ion concentrations of 0.20 and 0.18 ppm, respectively. These low‐leached ion concentrations further support the excellent cell viability results achieved for the indirect cytotoxicity tests.

On the other hand, immunofluorescence analysis was performed to detect the effect of direct exposures of TiAu, TiAuN, and TiAuO thin film surfaces on the cell morphology of L929 mouse fibroblasts (Figure 8d). Specifically, following exposures, L929 cells were stained with DAPI (nuclei) and Phalloidin (actin filaments). Once again, cells were also directly incubated with Cu substrates and DMSO 10% (used as positive controls), while exposure of cells to Ti‐6Al‐4 V substrates were used as negative controls. Our data revealed that exposures to both Cu substrates and 10% DMSO resulted in a significant alteration of cell morphology/shape as evidenced by a robust disruption of cell membrane integrity and alterations in actin filaments staining, suggesting a strong cytotoxic effect. Conversely, images obtained following thin films exposures, show that the morphology of cells is similar to DMEM (blank sample) and Ti‐6Al‐4 V substrate‐treated cells, in terms of normal staining for both nucleus and actin filaments staining, indicative of healthy L929 cells. The above data, under the same experimental conditions, were further supported through Alamar Blue analysis following direct exposures of L929 cells to the samples (Figure 8e). Specifically, cell viability levels after treatments were comparable and similar to those for the indirect extract exposure method (Figure 8b). In particular, viability levels were detected to be between 94% and 101% in exposures with both the thin films and Ti‐6Al‐4 V substrates, while once again Cu substrates and 10% DMSO were highly cytotoxic, causing a dramatic decrease of viable cells following 72 h of exposure. Overall, our results demonstrate the excellent biocompatibility potential of the TiAu, TiAuN, and TiAuO thin films against L929 fibroblast cells, as evidenced in both direct and indirect exposure protocols.

#### 2.3.2 Antibacterial Test Results

**Figure 9:**
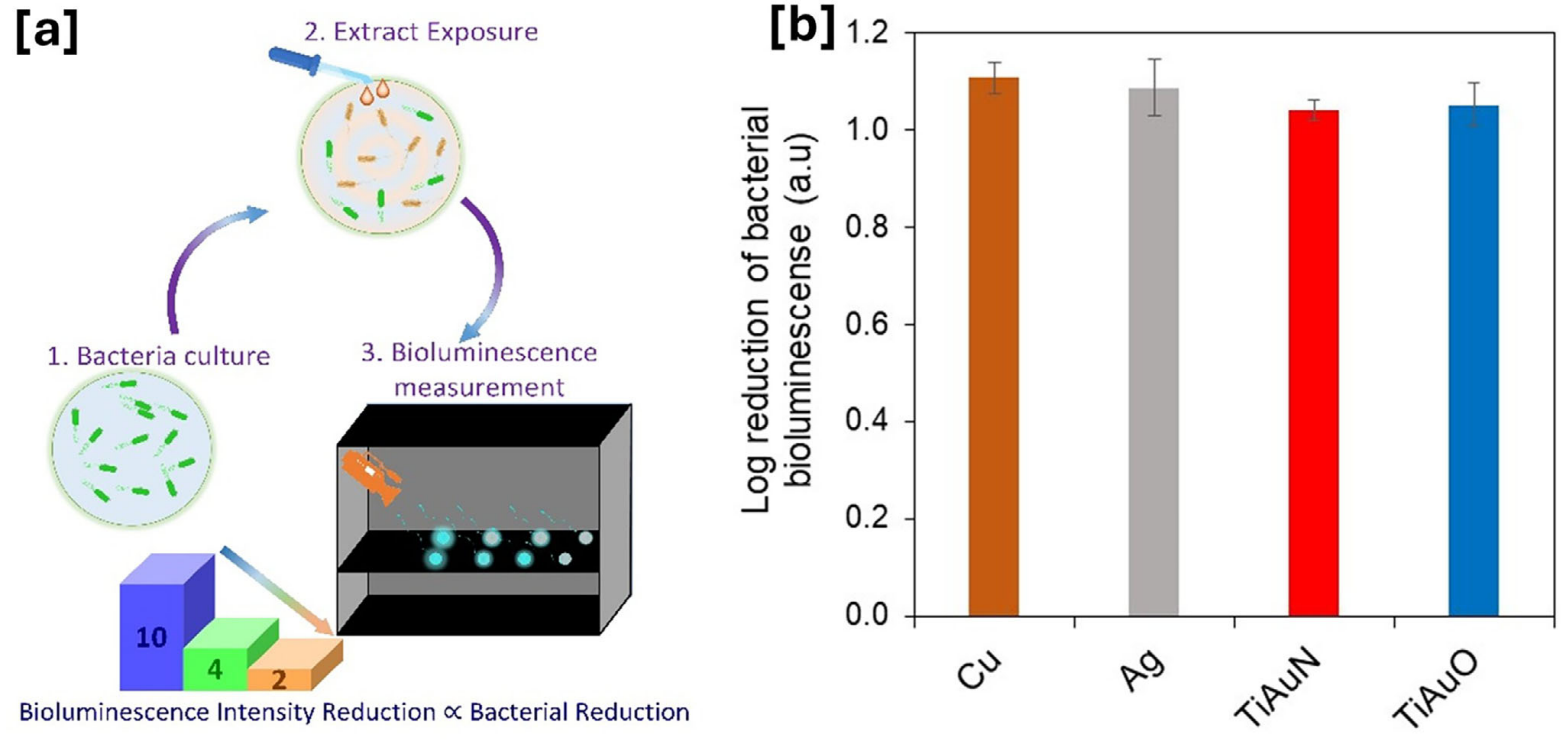
a) Schematic representation of the antibacterial test procedure using the bioluminescence output of *E. coli* pQE‐ilux bacteria as an index of death. b) Log reduction of bioluminescence response from *E. coli* bacterial colonies following 19 min of exposure to Cu, Ag, and TiAuN and TiAuO thin films. Data presented as mean ± SD, *n* = 3. Any statistically significant differences in results are indicated as *p < 0.05, **p < 0.01, and ***p < 0.001.

The antibacterial performance of the TiAuN and TiAuO thin film surfaces was analyzed using the bioluminescence output of *Escherichia coli* pQE‐ilux bacteria as an index of death. The number of live *E. coli* emitting bioluminescence following exposure to the sample surfaces was measured in a dark box and used as a reference to indicate antibacterial performance of the thin films (**Figure** **9**a). This assay is considered better than traditional recovery assays that equate recovery of live cells with death, as it also accounts for recovery artefact relating differential adhesion of the bacteria to the various surfaces. Moreover, our previous in vitro studies on Cu surfaces using this technique have shown excellent correlation between reduction in bioluminescence and *E. coli* death. Results for the logarithmic reduction in live *E. coli* emitting bioluminescence after 19 min of exposure to TiAuN and TiAuO thin film surfaces are presented in Figure 9b together with the results for Cu and Ag control surfaces, which are well‐established antimicrobial agents.^[^ ^3^ ^]^ The results for all four surfaces are very similar with no statistically significant differences present. The Cu control surface presents strong antibacterial behavior with a 1.11 log reduction in bioluminescence in <20 min test period, while the Ag control surface gives a similar 1.09 log reduction. The results for the TiAuN and TiAuO thin films are also similar to the Cu and Ag control surfaces, with log reductions of 1.04 and 1.05, respectively. This strong antibacterial activity is provided by the significant Au contents of 20.6 and 22.3 at.% in these two coatings. Like Cu and Ag, Au is another element that has been previously used in combination with other agents to impart antimicrobial activity to sputtered metal‐based thin film coatings for biomedical applications.^[^ ^39^ ^]^ The strong antibacterial effect of surfaces containing Cu, Ag, and Au has been attributed to a combination of damage inferred by metal ions and reactive oxygen species (ROS), leading to lipid peroxidation and subsequent loss of membrane integrity, protein damage, DNA and RNA damage and cell death.^[^ ^3^, ^40^ ^]^ While this study has confirmed the antibacterial potential of TiAuN and TiAuO thin film surfaces, further detailed analysis is required to understand its exact bacteria‐killing mechanism.

## 3 Conclusion 

This work demonstrates the first successful attempt to grow nitrogen and oxygen‐doped Ti_3_Au thin film structures with increased dislocation slip plane energy and α‐phase stability to achieve exceptional biotribological and antibacterial performance. These new Ti_3_Au:N and Ti_3_Au:O thin film coatings were grown to a thickness of ≈1 µm on Ti‐6Al‐4 V substrates by magnetron co‐sputtering of Ti and Au in varying Ar:N_2_ and Ar:O_2_ gas environments at 450 °C. The resulting coatings had grainy topography and columnar structures and were highly crystalline, containing α stabilizing phases of Ti_3_Au. There was a significant increase in hardness for the thin films of ≈200–250% when compared to the bare Ti‐6Al‐4 V substrate, with a maximum value of 14.7 GPa achieved for the Ti_3_Au:N sample. A similar trend was observed for scratch resistance with increases in critical load of 3.5–4.5 times for the Ti_3_Au:N and Ti_3_Au:O coatings compared to the Ti‐6Al‐4 V substrate. Coating the bare Ti‐6Al‐4 V substrate with the new coatings also gave a significant improvement in wear resistance. The coefficient of friction reduced from 0.5 for the Ti‐6Al‐4 V substrate to values of 0.2, 0.1, and 0.09 for the Ti_3_Au, Ti_3_AuO, and Ti_3_Au:N coatings, respectively, which resulted in considerable 10‐to‐20‐fold reductions in wear rates. All coatings were found to be highly biocompatible, presenting extremely safe cytotoxic profiles during both direct and indirect tests with L929 mouse fibroblasts, with cell viability levels of >90% and leached ion concentrations <0.2 ppm following 168 h of exposure. The Ti_3_Au:N and Ti_3_Au:O coatings also demonstrated strong antibacterial potential against *E. coli*, with similar log reductions in bacterial bioluminescence as established biocidal Cu and Ag elements. Overall, this work demonstrates a new class of advanced biocompatible Ti_3_Au:N and Ti_3_Au:O coatings with outstanding multifunctional biotribological performance. Their combination of high hardness, scratch resistance, low friction, and antibacterial properties positions them as a breakthrough material for medical implants and devices requiring both mechanical durability and infection resistance

## 4 Experimental Section

### Thin Film Deposition

The Ti_3_Au:N and Ti_3_Au:O thin films were deposited using a Moorfield Nano‐PVD magnetron sputtering system. Two individual sputtering targets of 50.4 mm diameter made from 99.99% pure Ti and Au, supplied by PI‐KEM Ltd, UK, were loaded onto DC and RF magnetrons, respectively (Figure 1a). Grade 5 titanium alloy (Ti‐6Al‐4 V) substrates, together with standard microscopic glass slides, both 1 mm thick, were prepared and cleaned as described in the previous work,^[^ ^19^ ^]^ before being loaded into the deposition chamber at a distance of 100 mm from the targets. The substrates were rotated at a constant speed of 5 rpm, and the chamber was evacuated to a base pressure better than 0.3 mPa. The substrates were then heated to a surface temperature of 450 °C for 40 min prior to the start of deposition to improve crystallization of the Ti_3_Au intermetallic during deposition.^[^ ^19^ ^]^ Following this, the chamber was made either inert or reactive by the introduction of argon (Ar) process gas or a mixture of Ar and nitrogen (N) or oxygen (O). The N and O gas flow rates were set at 0.7 sccm, while the Ar flow rate was set at 5 sccm to achieve a working pressure of 0.3 Pa, resulting in Ar:N and Ar:O ratios of 7:1. The power levels on the Ti and Au magnetrons were individually controlled at 321 and 31 W, respectively, to achieve a Ti:Au stoichiometry close to the required value of 3:1. The targets were then pre‐sputtered for 300 s to remove surface contaminants and stabilize the plasma before the target shutters were opened for 60 min to perform co‐deposition of Ti and Au under inert (Ar) or reactive (Ar/N_2_, Ar/O_2_) environments and achieve a total film thickness in the region of 1 µm. Following deposition, the thin films were allowed to cool under vacuum to a temperature lower than 50 °C to prevent excessive oxidation, before being removed from the chamber. The resulting three Ti_3_Au, Ti_3_Au:N, and Ti_3_Au:O thin film samples were denoted with identifications of TiAu, TiAuN, and TiAuO, respectively (Figure 1b).

### Structural, Chemical, and Morphological Characterization

The crystalline nature of the deposited TiAu, TiAuN, and TiAuO thin films were analyzed by the X‐ray diffraction (XRD) technique using a Rigaku SmartLab SE X‐ray diffractometer, equipped with Cu K_α_ radiation, in parallel beam (θ–2θ) configuration. The diffraction patterns were collected over a 2θ range of 10°–80° and the generated peaks were indexed using the inbuild crystallographic database and verified with standard collection files from the ICSD (Inorganic Crystal Structure Database). The surface topology of the sample surfaces and cross‐section regions were studied from high‐resolution images generated from a Tescan MIRA 3 scanning electron microscope (SEM), operating at 5 kV for imaging. Elemental composition of each sample was verified using an Oxford Instruments X‐Max 150 energy dispersive X‐ray (EDX) spectroscopy detector attached to the SEM. Elemental depth profiling through the film thickness was performed using a Hiden Analytical secondary ion mass spectrometer (SIMS) with an Ar gas ion gun rastered over a 500 × 500 µm area and a quadruple detector. Transmission electron microscope (TEM) lamellae were prepared by focused ion beam (FIB) milling using a FEI Helios Nano Lab 600 Dual Beam system, outfitted with a focused 30 keV Ga liquid metal ion source. TEM imaging was obtained with a JEOL 2100F TEM operated at 200 KeV and micro‐analysis was performed at 200 KeV, 0.7 nA in scanning transmission operation using an Oxford Instruments micro‐analysis system. A 3D profile of 3 × 3 µm area was created using a Veeco Dimension 3100 atomic force microscope (AFM) fitted with a 15 nm sharp silicon tip operating in continuous contact mode, to understand the variation in surface roughness.

### Mechanical, Tribological, and Electrochemical Characterization

Mechanical hardness and elastic modulus of the TiAu, TiAuN, and TiAuO thin films were measured by performing nanoindentation using a three‐sided Berkovich tip in a Bruker–Hysitron TI900‐Triboindenter nanomechanical testing system. A set of 16 indentations in a 4 × 4 pattern, with 10 µm spacing between each indent, was made on each sample. Substrate effects were avoided by limiting the indentation depth to 10% of the total film thickness. The load‐displacement curve from each indentation was plotted and the unloading curve was analyzed according to the Oliver and Pharr method to extract the mechanical performance. Scratch resistance was measured using a Nanovea PB1000 mechanical tester with a sphero‐conical 120° radius 10 µm diamond tip operated in progressive load mode from 0.01 to 2 N at a speed of 4 mm min^−1^ over a scratch length of 2 mm. Friction and wear behavior were evaluated using a Nanovea T50 tribometer with AISI 440 stainless steel balls of 6 mm diameter sliding against the coating samples submerged in simulated body fluid (SBF)^[^ ^41^ ^]^ at room temperature. A normal load of 1.52 N was applied to the stainless‐steel ball when sliding in linear reciprocal motion mode at an amplitude of 5 mm and frequency of 1 Hz for 20 min, giving a total sliding distance of 12 m. Following the scratch and wear tests, the scratch tracks and wear scar morphologies were examined using the Nanovea PB1000 integrated optical microscope, with coaxial white light illumination, and the wear volume was measured using a non‐contact Nanovea JR25 profilometer.

### Biocompatibility Characterization—Cells and Reagents

In the current study, L929 murine fibroblast cells were used as biological material, which were purchased from Deutsche Sammlung von Microorganismen and Zellkulturen (DSMZ − Braunschweig, Germany). L929 cells were cultured as previously described.^[^ ^20^, ^21^ ^]^ Cell culture media, antibiotics, fetal bovine serum (FBS), trypsin, and Phosphate Buffer Saline (PBS) were purchased from Biosera (USA), while Resazurin sodium salt was obtained from Fluorochem (UK). In addition, nucleus staining reagent 4′,6‐diamidino‐2‐phenylindole (DAPI) was from Sigma–Aldrich Co. (Germany), Phalloidin CF440 antibody was purchased from Biotium (USA), while fluorescent mounting medium (S3023) was from DAKO (Denmark). Finally, cover glasses were from Paul Marienfeld GmbH & Co. KG (Germany).

### Biocompatibility Characterization—Preparation of Thin Film Extracts

In order to investigate the in vitro cytotoxic effect of the TiAu, TiAuN, and TiAuO thin films against L929 fibroblasts, two distinct extraction protocols were performed in terms of ion leaching. Specifically, in the first set of extracts preparation protocol, thin films (4 × 4 mm) were immersed into plastic petri dishes, containing 2 mL of DMEM culture media and were incubated for 72 h, in a humidified incubator at 37 °C and 5% CO_2_. In parallel, in the second set of extracts preparation from the same samples, following the initial 72 h ion leaching period, the immersion period of the thin films was extended for additional 96 h (168 h in total). In both sets of extracts preparation, Cu and Ti‐6Al‐4 V substrates were used as positive and negative controls, respectively, while petri dishes were slightly (5 s) agitated, at the beginning and the end of extraction periods, to increase ion leaching to the media.

### Biocompatibility Characterization—Biocompatibility of Thin Films Against L929 Cell Line—Indirect Biocompatibility

Following the completion of both ion leaching extraction protocols, the in vitro biocompatibility/cytotoxicity of undiluted TiAu, TiAuN, and TiAuO thin film extracts against L929 fibroblasts was determined by the Alamar Blue assay and ion leaching potential as per ISO 10993–5/USP 87 standards. Initially, L929 mouse fibroblast cells were plated at a density of 2000 cells per well into a 96‐well culture plate and left to incubate overnight at 37 °C and 5% CO_2_. The next day DMEM culture media was removed and replaced with culture media containing ions from both 72 and 168 h leaching periods. In each exposure protocol, fibroblasts were incubated with both types of leached extracts for a total period of 72 h. In parallel, cells were also exposed to ion‐leached extracts obtained from Cu (positive control) and Ti‐6Al‐4 V (negative control) substrates. Accordingly, L929 cells were also incubated with 10% DMSO (positive control) and DMEM culture media (Blank samples).

### Biocompatibility Characterization—Biocompatibility of Thin Films Against L929 Cell Line—Direct Biocompatibility

In another set of experiments, the direct biocompatibility of thin films against L929 fibroblast cells was assessed without performing any leaching procedures. Specifically, TiAu, TiAuN, and TiAuO thin films were directly inserted into 96‐well plates, previously seeded with 2000 cells mL^−1^ of fibroblast cells and incubated for a total period of 72 h. Similarly, in this set of experiments, Cu substrates and 10% of DMSO were used as positive controls, while for negative control conditions, fibroblasts were treated with Ti‐6Al‐4 V substrates and DMEM culture media (Blank samples). Following the completion of both indirect and direct exposures of L929 cells for 72 h with ion‐leached extracts obtained from the thin films’ extraction protocols and thin films immersion directly to 96‐well plates, respectively, cell viability was determined through the Alamar Blue assay as previously described.^[^ ^19^, ^22^ ^]^ Finally, viability levels were expressed as a percentage compared to untreated (Blank) cells. Ions leached from the TiAu, TiAuN, and TiAuO thin films and Cu and Ti‐6Al‐4 V substrates into the extracts were measured with a Perkin Elemer Optima 8000 inductively coupled plasma optical emission mass spectrometer (ICP‐OEMS), while the results were compared to a set of calibrated standards containing 0.625, 2.5, 5 and 10 ppm of dissolved Ti, Al, V, Cu and Au.

### Biocompatibility Characterization—Biocompatibility of Thin Films Against L929 Cell Line—Immunofluorescence Analysis

For the purposes of immunofluorescence analysis, mouse fibroblasts cells were seeded on glass coverslips previously inserted into petri dishes and incubated overnight at 37 °C and 5% CO_2_. The next day, TiAu, TiAuN, and TiAuO thin film samples were added into the petri dishes and L929 cells were directly incubated for 72 h. Accordingly, mouse fibroblasts were incubated with Ti‐6Al‐4 V substrates (negative controls) and Cu substrates as well as 10% of DMSO in culture media (positive controls). At the end of exposures, following PBS washes, cells were fixed on ice for 15 min with 3.75% formaldehyde in PBS. After a second set of PBS washes, L929 cells were permeabilized for 10 min through the addition of PBS (pH 7.4), containing 0.5% Triton‐X for 10 min. Cells were subsequently washed three times with PBS and incubated for 20 min at room temperature, with fluorescent Phalloidin antibody. Next, cells were washed once again with PBS and cell DNA was counterstained with DAPI (1 µg mL^−1^) for 5 min. Finally, following three PBS washes, coverslips were mounted with fluorescent mounting medium prior to observation. For the acquisition of cell images, a Zeiss fluorescence microscope with 40x lens was used (Zeiss Axionvision software, Carl Zeiss Microimaging, Oberkochen, Germany), while image analysis was performed with ImageJ software.

### Antibacterial Test

*E. coli* TOP10 cells expressing the recombinant *ilux* gene cassette^[^ ^42^ ^]^ were cultured aerobically at 37 °C in LB broth containing a final concentration of 100 µg mL^−1^ Ampicillin (Amp). The cells were grown for 24 h before centrifugal harvesting at 3200 g for 15 min, followed by resuspension washing in 10 mL of fresh LB/Amp. The centrifugation cycle was then repeated to produce a pellet of cells, which was resuspended in 5 mL of fresh LB/Amp. The cells were then divided into 5 µL aliquots at a concentration of 6 × 10^9^ colony‐forming units per milliliter (CFU/mL) for the antibacterial tests. The TiAu, TiAuN, and TiAuO thin films were attached to a glass plate using adhesive tape and the 5 µL aliquots of cell suspension were then deposited on the center of each film samples. The films were then transferred to a non‐illuminated GeneSys (Syngene) GBox with GeneTools (Syngene) software and images of the individual 5 µL droplets were taken after 1, 4, 7, 10, 13, 16, and 19 min of exposure. The resulting TIFF images were then visually analyzed to identify bioluminescent dots that were then individually selected using the software selection tool with an appropriate background sample acting as a blank reading. The background light intensity of the individual dots was then removed, and experimental triplicates were used to calculate logarithmic values of average bioluminescence for each exposure time.

### Statistical Analysis

The data were presented as mean ± standard deviation (SD). The statistical analyses were performed using a one‐way analysis of variance (ANOVA), followed by Tukey's post‐hoc test for multiple group comparisons. The thresholds for statistical significance were set at ^*^ *p* < 0.05, ^**^ *p* < 0.01, and ^***^ *p* < 0.001. The statistical analyses were performed using GraphPad Prism 10 (GraphPad Software, San Diego, CA, USA).

## Conflict of Interest

The authors declare no conflict of interest.

## Author Contributions

C.C.L. conducted investigation, formal analysis, validation, data curation, visualization, and writing—original draft. I.A. conducted investigation, formal analysis, validation, and writing—review & editing. I.S.P. conducted investigation, formal analysis, and validation. G.Z. contributed to methodology, investigation, formal analysis, validation, and writing—review & editing. A.M.B. conducted investigation, formal analysis, validation, and writing—review & editing. L.G.D. contributed to methodology, investigation, formal analysis, validation, and writing—review & editing. L.B. contributed to methodology, investigation, and formal analysis. A.S.A. contributed to methodology, investigation, formal analysis, validation, and writing—review & editing. L.M. contributed to methodology, investigation, formal analysis, validation, and writing—review & editing. D.M. contributed to methodology, investigation, formal analysis, validation, and writing—review & editing. M.I.P. contributed to conceptualization, methodology, writing—review & editing, supervision, and funding acquisition. M.B. contributed to conceptualization, methodology, writing—original draft, supervision, project administration, and funding acquisition. All authors commented on the paper and approved the final version of the manuscript.

## Ethics Approval and Consent to Participate

This study did not involve the use or participation of animals or human subjects.

## Data Availability

The data that support the findings of this study are available from the corresponding author upon reasonable request.
